# Modified suture-assisted penetrating canaloplasty vs. compound trabeculectomy in treating primary angle-closure glaucoma

**DOI:** 10.1186/s12886-025-04421-0

**Published:** 2025-10-21

**Authors:** Jianhui Zhang, Ningning Lin, Liu Zhang, Shancheng Si

**Affiliations:** 1 School of Clinical Medicine, Eye institute and Affiliated Xiamen Eye Center of Xiamen University, Xiamen University, Xiamen, 361004 China; 2 Fujian Provincial Key Laboratory of Corneal & Ocular Surface and Corneal Diseases, Xiamen, Fujian 361002 China; 3 Department of Ophthalmology, Liaoning Chaoyang Central Hospital, Chaoyang, 122000 China; 4 Department of Glaucoma, Fuzhou Eye Hospital, Fuzhou, 350007 China; 5 Beijing Tsinghua Changgung Hospital Eye Center, Beijing Visual Science and Translational Eye Research Institute (BERI), Tsinghua Medicine, Tsinghua University, 168 Litang Road, Changping District, Beijing, 102218 P.R. China

**Keywords:** Suture-assisted penetrating canaloplasty, Compound trabeculectomy, Primary angle-closure glaucoma, Canaloplasty

## Abstract

**Purposes:**

To evaluate the effectiveness and safety of a modified suture-assisted penetrating canaloplasty (SAPC) compared to compound trabeculectomy for treating primary angle-closure glaucoma (PACG).

**Design:**

Retrospective comparative interventional study.

**Methods:**

We analyzed consecutive cases of uncontrolled PACG treated at Fuzhou Eye Hospital (December 2019-April 2021) with either modified SAPC (study group) or compound trabeculectomy (control group). Postoperative evaluations occurred at 1 week and 1, 3, 6, and 12 months. Outcome measures included mean diurnal intraocular pressure (mdIOP), anti-glaucoma medication burden, surgical success rate (complete: mdIOP ≤18 mmHg without medications; qualified: mdIOP ≤18 mmHg with medications), and complications.

**Results:**

The final analysis included 19 eyes in the modified SAPC group and 17 eyes in the control group. At the 12-month follow-up, the SAPC group demonstrated a significant reduction in mdIOP from 20.17±6.00 mmHg to 12.38±2.60 mmHg (P<0.001), with medications reduced from 3.21±1.03 to 0.11±0.32 (P<0.001). Compared to the control group's mdIOP of 16.82±2.36 mmHg with 0.43±0.68 medications at 12 months, the modified SAPC group maintained significantly better pressure control (P<0.001). The complete success rate was substantially higher in the SAPC group (89.47% vs. 52.94%, P=0.038). Transient IOP elevation occurred in 5 eyes (26.3%) following SAPC, representing the most frequent postoperative complication. No significant vision loss or serious adverse events were observed.

**Conclusion:**

Modified SAPC provides effective and sustained IOP reduction with a favorable safety profile, outperforming compound trabeculectomy in PACG patients over 12 months. while minimizing medication dependence, demonstrating superior effectiveness to compound trabeculectomy with a favorable short-term safety profile.

**Key Summary Points:**

Why carry out this study?

1. This study aimed to evaluate the effectiveness and safety of a modified suture-assisted penetrating canaloplasty (SAPC) technique compared to compound trabeculectomy in treating primary angle-closure glaucoma (PACG).
2. This study addressed the limitations of high-cost microcatheters and bleb-related complications.

What was learned from the study?

1. Modified SAPC demonstrated superior intraocular pressure (IOP) reduction (12.38±2.60 mmHg vs. 16.82±2.36 mmHg at 12 months) and higher complete success rates (89.47% vs. 52.94%) compared to compound trabeculectomy, with fewer complications and reduced dependence on postoperative medications.
2. The technique’s cost-effective use of 5-0 polypropylene sutures and adjustable sutures simplified the procedure while maintaining high catheterization success (87.5%), offering a viable alternative for mild-to-moderate PACG.
3. Younger patients with short axial lengths showed an increased risk of malignant glaucoma post-SAPC, highlighting the need for refined surgical approaches in this subgroup.

## Introduction

Originally developed as a bleb-independent procedure for primary open-angle glaucoma (POAG), canaloplasty enhances physiological aqueous inner outflow by mechanically tensioning the suture-dilated Schlemm’s canal (SC) and collecting ducts instead of external drainage through the filtering bleb, thereby circumventing the bleb-related complications associated with compound trabeculectomy. Building upon this concept, Liang et al. pioneered penetrating canaloplasty, which integrates canaloplasty with trabeculectomy principles. This innovative approach creates direct communication between the anterior chamber (AC) and SC ostia through a surgically created window, significantly expanding its applicability to various glaucoma subtypes, including primary angle-closure glaucoma (PACG). [1, 2]

While iTrack microcatheters have become standard in canaloplasty, their high cost imposes significant financial burdens on patients. Although some researchers have attempted to substitute them with prefabricated double-helical 6 − 0 sutures, this alternative remains technically challenging[3].

To address these limitations while minimizing bleb-related complications associated with compound trabeculectomy (the current gold standard for progressive glaucoma [4]), we developed a modified suture-assisted penetrating canaloplasty (SAPC) technique. Our innovation utilizes 5 − 0 polypropylene sutures combined with a prefabricated adjustable suture system, significantly simplifying the procedure while enhancing surgical outcomes. The following report compares the effectiveness and safety profiles of this novel SAPC approach against compound trabeculectomy.

## Methods

### Participants and preoperative assessments

This interventional case-control study enrolled patients with uncontrolled PACG who underwent either modified SAPC or compound trabeculectomy at Fuzhou Eye Hospital between December 2019 and April 2021. All SAPC procedures were performed by a single experienced surgeon (Z. J.) in the Department of Ophthalmology. The study protocol received ethical approval from the Institutional Review Board of Fuzhou Eye Hospital (approval no. FZYKYY-KY-2023-004) and adhered to the principles of the Declaration of Helsinki. All participants provided written informed consent, and data were anonymized prior to analysis.

Eligible participants met the following criteria: diagnosis of PACG (age ≥ 18 years)[5, 6], intraocular pressure (IOP) > 21 mmHg without anti-glaucoma medications (regardless of prior laser iridotomy), intolerance/allergies to multiple topical anti-glaucoma agents or documented nonadherence, ≥ 180 degrees of angle closure on gonioscopy, and compromised corneal endothelial function (a corneal endothelial cell density of < 1500 cells/mm²) precluding safe phacoemulsification or lacking lens removal indications. We excluded patients with POAG or secondary glaucoma, axial length ≤ 20 mm, previous glaucoma surgery, best-corrected visual acuity (BCVA) ≤ hand motion (HM) or visual field mean defect ≤−12 dB, active psychiatric conditions, current anti-coagulation therapy, significant systemic comorbidities, or contraindications to anti-glaucoma surgeries.

### Grouping and examinations

**Fig. 1:**
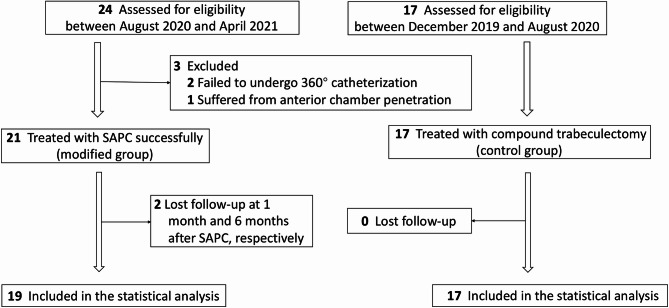
Flow chart of recruitment and grouping of study eyes. SAPC, suture-assisted penetrating canaloplasty

 The modified SAPC technique was initially developed in August 2020. Given its promising effectiveness in preliminary cases, all eligible patients enrolled between August 2020 and April 2021 underwent this procedure (study group), whereas those meeting the same inclusion criteria during the earlier period (December 2019–August 2020) received compound trabeculectomy (control group). Notably, eyes initially assigned to the study group were reallocated to the control group if 360° catheterization failed intraoperatively (Fig. 1). Although non-randomized, the study adhered to a uniform design with standardized inclusion/exclusion criteria and outcome definitions (success/failure). All participants were of Han Chinese ethnicity.

Clinical data collection included: demographic characteristics (age, sex); BCVA at baseline and 12 months postoperatively; mean diurnal intraocular pressure (mdIOP, averaged from three daily measurements) and slit-lamp findings at baseline, 1 week, and 1, 3, 6, and 12 months; ultrasound biomicroscopy (UBM) at baseline; anterior segment optical coherence tomography (AS-OCT) at 3, 6, and 12 months; preoperative gonioscopy, non-mydriatic fundus photography, and retinal nerve fiber layer thickness by OCT. Medication use and complications were recorded at all visits, with additional bleb monitoring in the control group. To minimize bias, mdIOP measurements were performed by a single researcher (Z. L.) masked to surgical assignment.

### Surgical procedures of SAPC

**Fig. 2:**
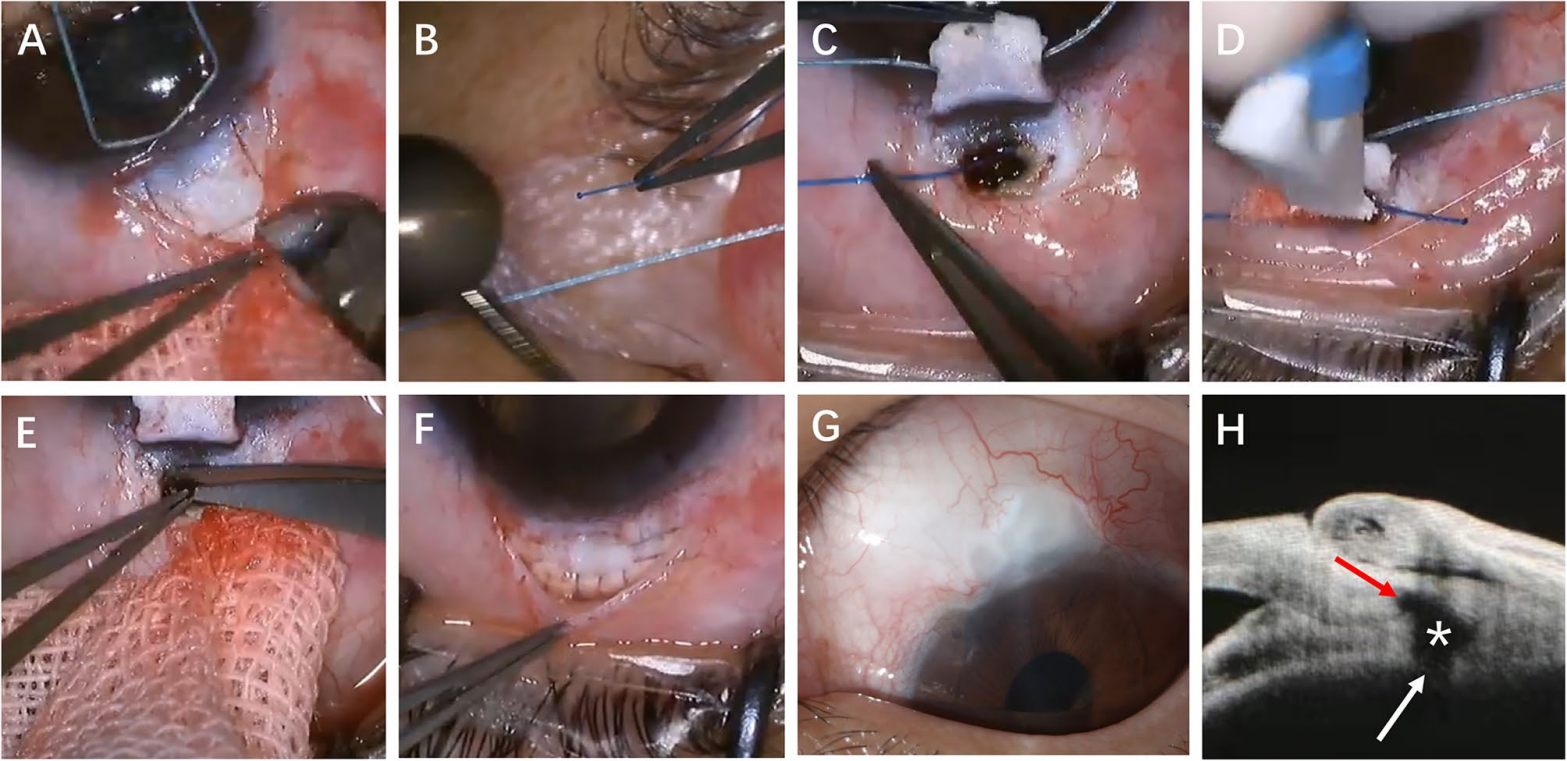
Surgical procedure of SAPC in eyes with PACG. **A** A superficial scleral flap with a square shape (about 4.0 mm × 4.0 mm) and about half the whole scleral thickness was sculpted in the 12 o’clock position.**B** The broken end of 5-0 polypropylene suture was heated into a spherical probe to replace the iTrack microcatheter. **C**. 360° catheterization with the 5-0 suture-probe was successfully completed after the outer wall of the SC was unroofed.**D** The 10-0 polypropylene tensioning suture was tied to the tip of the 5-0 suture-probe and then introduced into the SC with the withdrawal of the probe. **E** Peripheral iridectomy was performed after a 1 mm × 0.5 mm piece of limbus tissue was cut off forward to the tensioning suture. **F** The superficial scleral flap was tightly sutured. **G** Six months after SAPC, no functional filtering bleb was formed, and the IOP was maintained at 12-15mmHg. **H** The AS-OCT 1 year after SAPC showed dilated SC (red arrow), scleral cistern (white asterisk) and trabecular fistula (white arrow). AS-OCT, anterior segment optical coherence tomography; IOP, intraocular pressure; PACG, primary angle-closure glaucoma; SAPC, suture-assisted penetrating canaloplasty; SC, Schlemm’s canal

All surgical procedures were performed by a single experienced surgeon (Z. J.) under retrobulbar anesthesia, as illustrated in Fig. 2. Preoperatively, IOP was carefully controlled to approach normal physiological levels to optimize surgical safety. The SAPC surgical technique began with creation of a fornix-based conjunctival flap at the 12 o’clock position. Two 0.2 mg/ml mitomycin-C (MMC)-soaked sponges (0.02%) were then placed beneath Tenon’s capsule for 3 min, positioned away from the limbus. Following sponges’ removal, thorough irrigation with 10 ml balanced salt solution was performed. Subsequent steps involved meticulous dissection of scleral flaps as previously described for Ab externo SC surgery[7].

1. A superficial square flap (4.0 × 4.0 mm) comprising approximately half scleral thickness.
2. A deeper rectangular flap (2.0 × 1.0 mm) leaving a thin translucent scleral layer overlying the choroid, extended 1–1.5 mm into clear cornea.

After unroofing SC and excising the deep flap, the surgeon fashioned a suture probe by modifying a 5 − 0 polypropylene suture with a unipolar electrocoagulation device to create a globular tip. Both SC ostia were dilated with viscoelastic to facilitate smooth probe insertion in either rotational direction.

AC deepening and anterior chamber angle (ACA) adhesion separation were achieved with viscoelastic injection. The modified probe was introduced into one SC ostium, with its passage through the canal confirmed either by direct gonioscopic visualization (using Ocular Ahmed DVX Surgical Gonio) or, in cases of complete angle closure/poor corneal transparency, by observing limbal bulge movement. A critical technical modification involved:

1. Securing a 10 − 0 polypropylene suture to the probe’s spherical tip upon its emergence from the contralateral ostium.
2. Withdrawing the 5 − 0 probe to thread the 10 − 0 suture through SC.
3. Creating a circumferential tensioning suture after disconnection.

The procedure concluded with excision of anterior SC and corneoscleral tissue (1 × 0.5 mm) using trabecular scissors, peripheral iridectomy, watertight scleral flap closure with 10 − 0 nylon, and conjunctival closure with interrupted 10 − 0 nylon sutures.

### Definations and main evaluations

The study employed standardized definitions for surgical techniques and outcome measures. Compound trabeculectomy was characterized as trabeculectomy augmented with intraoperative peripheral iridectomy and application of 0.02% MMC. Two distinct success criteria were established: Criterion 1 required postoperative mdIOP between 5 and 21 mmHg with ≥ 30% reduction from baseline, while Criterion 2 maintained stricter control (5–18 mmHg) with identical percentage reduction. Both criteria differentiated complete success (achieved without medications) from qualified success (requiring adjunctive therapy)[2, 8, 9]. Surgical failure was determined by either: (1) two consecutive mdIOP measurements > 21 mmHg despite maximal tolerated medical therapy after adjustable suture removal and ocular massage (≥ 1 month postoperatively), or (2) light perception loss attributable to surgical complications. Early postoperative complications were categorized as transient hypotony (mdIOP < 5 mmHg) or transient IOP elevation (mdIOP > 21 mmHg) within three months. Postoperative evaluation included comprehensive anterior segment assessment using slit lamp examination and UBM to verify filtering bleb formation and functionality. For detailed anatomical analysis, either UBM or AS-OCT was employed to precisely evaluate SC ostium morphology and the degree of canal distension achieved through the tensioning suture technique. Baseline IOP measurements were obtained following complete cessation of all ocular hypotensive medications prior to surgical intervention.

### Statistical analysis

 Patients with less than one year of follow-up were excluded from final analysis. All statistical computations were conducted using SPSS Statistics (version 25.0, IBM Corp.). Continuous variables were expressed as mean ± standard deviation (SD) for normally distributed data or median (range) for non-parametric distributions, while categorical variables were presented as frequencies and percentages. Data normality was verified using the Shapiro-Wilk test.

Between-group comparisons of mdIOPs at each timepoint employed independent samples t-tests, whereas within-group mdIOP changes from baseline were analyzed using repeated-measures analysis of variance (ANOVA). The Mann-Whitney U test evaluated differences in anti-glaucoma medication requirements both longitudinally (pre- vs. postoperative) and cross-sectionally (study vs. control groups). Categorical variables were compared using χ² or Fisher’s exact tests as appropriate. Visual acuity outcomes (proportions with BCVA > HM and ≤ 20/400) were assessed via McNemar’s test.

Success rates were illustrated using Kaplan-Meier survival curves, with between-group differences examined through log-rank testing. A two-tailed P-value < 0.05 defined statistical significance for all analyses.

## Results

Twenty-four eyes (17 patients) were initially enrolled for the modified SAPC procedure. Three eyes required conversion to compound trabeculectomy due to technical challenges (two failed 360° catheterization attempts, and one suffered from AC penetration), resulting in 21 eyes (87.5%) successfully completing the planned SAPC with circumferential catheterization. Two eyes from separate patients were lost to follow-up at 1- and 6-month intervals. The final analysis included 19 eyes from 14 participants (5 females, 9 males; mean age 57.53 ± 12.25 years), all demonstrating complete surgical success at 12-month evaluation. Postoperative imaging (UBM/AS-OCT) confirmed direct aqueous communication between the ACA and SC without significant bleb formation in these cases. The control group comprised 17 eyes from 17 participants (11 females, 6 males; mean age 63.41 ± 4.40 years) who underwent standard compound trabeculectomy (Fig. 1). Baseline characteristics showed no significant differences between groups (all *P* > 0.05).

Notably, comparative analysis revealed borderline statistical differences in mean age (*P* = 0.063), preoperative mdIOP (20.17 ± 6.00 vs. 18.00 ± 3.24 mmHg, *P* = 0.074), and non-glaucomatous optic cup prevalence (36.8% vs. 64.7%, *P* = 0.095) between the groups (Table 1).

**Table 1:** Demographic information of the participants and baseline characteristics of operated eyes

Baseline characteristics	Grouping		*P* value
	Study	Control	
No. of participants	*N* = 14	*N* = 17	
Male	9 (64.29)	6 (35.29)	0.108
Age, y	57.53 ± 12.25	63.41 ± 4.40	0.063
No. of operated eyes	*N* = 19	*N* = 17	
Preoperative mdIOP, mmHg	20.17 ± 6.00	18.00 ± 3.24	0.074
No. of preoperative ocular hypotensive eye drops	3 (2, 5)	4 (2, 4)	0.361
Non-glaucomatous optic cup	7 (36.84)	11 (64.71)	0.095
360° angle-closure on UBM	8 (42.11)	11 (64.71)	0.175
Preoperative paracentesis of AC	0 (0)	3 (17.65)	0.191
History of IOL implantation	4 (21.05)	1 (5.88)	0.406
History of YAG-LPI	1 (5.26)	0 (0)	1.000
Pre-BCVA distribution of operated eyes			0.229
>HM and ≤ 20/400	4 (21.05)	1 (5.88)	
>20/400 and ≤ 20/60	4 (21.05)	8 (47.06)	
>20/60 and ≤ 20/30	6 (31.58)	6 (35.29)	
>20/30 and ≤ 20/20	5 (26.32)	2 (11.76)	

*AC* Anterior chamber, *BCVA* Best corrected visual acuity, *IOL* Intraocular lens, *LPI* Laser peripheral iridectomy, *mdIOP* The mean diurnal intraocular pressure, *UBM *Ultrasound biomicroscope, *YAG* Yttrium aluminium garnet

Data were presented as mean ± standard deviation, median (range) or no. (%)

*P* < 0.05 was considered to be statistically significant

At 12 months, all SAPC eyes achieved the primary success endpoint (mdIOP reduction > 30% without ocular hypotensive medication). While complete success rates (Criterion 1: mdIOP ≤ 21 mmHg) were comparable between groups (100% study vs. 94.12% control, *P* = 0.472), SAPC exhibited superior performance under the more stringent Criterion 2 (mdIOP ≤ 18 mmHg: 89.5% vs. 52.9%, *P* = 0.038). Qualified success rates reached 100% in both groups for Criterion 1 and 100% versus 76.47% for Criterion 2 (*P* = 0.087), with no surgical failures observed in either cohort.

The SAPC group demonstrated robust mdIOP reduction from preoperative levels (20.17 ± 6.00 mmHg on 3.21 ± 1.03 medications) to 12.38 ± 2.60 mmHg on 0.11 ± 0.32 medications at 12 months (both *P* < 0.001). Significant pressure reductions were maintained throughout follow-up, with mdIOP measurements of 14.22 ± 5.20 mmHg (1 week), 13.66 ± 7.30 mmHg (1 month), 11.66 ± 2.84 mmHg (3 months), and 11.39 ± 2.82 mmHg (6 months) (all *P* < 0.001 versus baseline). In contrast, the control group showed modest mdIOP changes from 18.00 ± 3.24 mmHg (preoperative, 3.43 ± 0.68 medications) to 16.82 ± 2.36 mmHg (12 months, 0.43 ± 0.68 medications; *P* = 0.262 for mdIOP, *P* < 0.001 for medications), with only transient early reductions at 1 week (12.97 ± 3.50 mmHg) and 1 month (14.43 ± 2.88 mmHg) achieving statistical significance (both *P* < 0.05). BCVA outcomes remained stable in both groups, with no significant differences in the proportions maintaining > HM and ≤ 20/400 vision (all *P* > 0.05, Table 2).

**Table 2:** The 1-year efficacy of modified SAPC vs. compound trabeculectomy in treating PACG

Follow-up variables	Grouping		*P* value
	Study (*N* = 19)	Control (*N* = 17)	
Each mdIOP			
Preoperative	20.17 ± 6.00	18.00 ± 3.24	0.074
Postoperative 1 week	14.22 ± 5.20^a^	12.97 ± 3.50^b^	0.318
Postoperative 1 month	13.66 ± 7.30^a^	14.43 ± 2.88^b^	0.603
Postoperative 3 months	11.66 ± 2.84^a^	16.47 ± 2.75	< 0.001**
Postoperative 6 months	11.39 ± 2.82^a^	16.43 ± 2.79	< 0.001**
Postoperative 12 months	12.38 ± 2.60^a^	16.82 ± 2.36	< 0.001**
***P*** value	<0.001**	<0.001**	
No. of ocular hypotensive eye drops			
Preoperative	3 (2, 5)	4 (2, 4)	0.361
Postoperative month 12	0 (0, 1)	0 (0, 3)	0.044*
*** P***value	<0.001	<0.001	
BCVA of > HM and ≤ 20/400			
Preoperative	4 (21.05)	1 (5.88)	0.130
Postoperative month 12	3 (15.79)	1 (5.88)	0.587
*** P***value	1.000	1.000	
Surgical results			
Complete success (criterion 1)	19 (100.00)	16 (94.12)	0.472
Qualified success (criterion 1)	19 (100.00)	17 (100.00)	-
Complete success (criterion 2)	17 (89.47)	9 (52.94)	0.038*
Qualified success (criterion 2)	19 (100.00)	13 (76.47)	0.087
Surgical failure	0 (0)	0 (0)	-

*ANOVA* Analysis of variance, *BCVA* Best corrected visual acuity, *HM* Hand movement, *mdIOP* The mean diurnal intraocular pressure, *PACG* Primary angle-closure glaucoma, *SAPC* Suture-assisted penetrating canaloplasty

* = *P* < 0.05

**= *P* < 0.001

Data were presented as mean ± standard deviation, median (range) or no. (%)

*P* < 0.05 was considered to be statistically significant.

^a^indicates that the difference is statistically lower compared with mdIOP before SAPC using repeated measured ANOVA

^b^indicates that the difference is statistically higher compared with mdIOP before compound trabeculectomy using repeated measured ANOVA

**Fig. 3:**
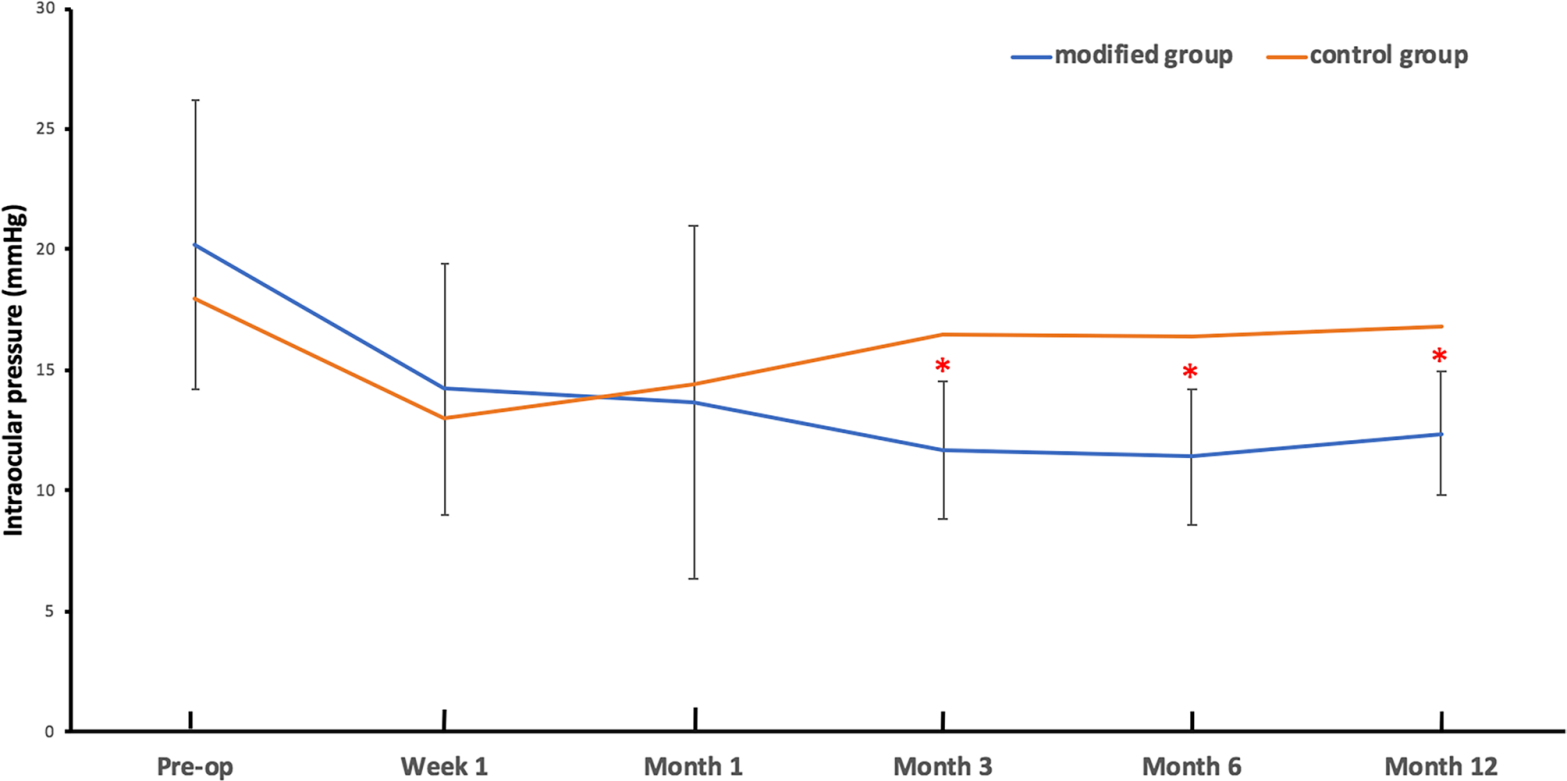
Follow up curves of IOP in two groups. Red asterisk represents that the IOP of the modified group receiving SAPC is statistically different from that of the control group using independent sample t-test. IOP, intraocular pressure; SAPC, suture-assisted penetrating canaloplasty

**Fig. 4:**
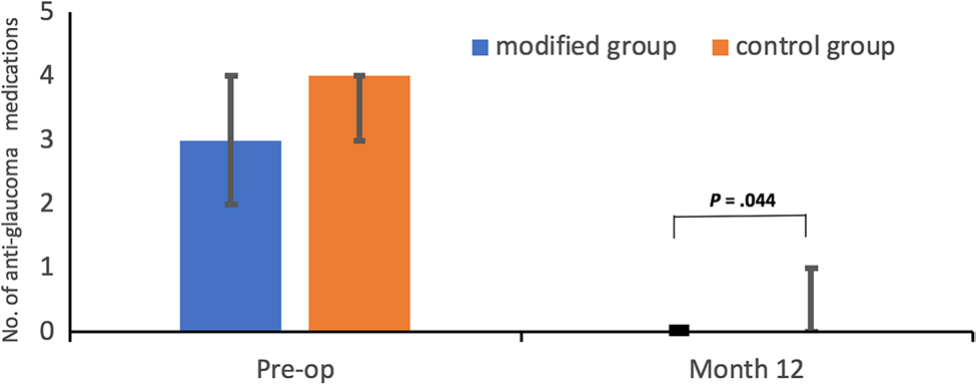
Histogram of anti-glaucoma medication changes preoperatively and 12 months after anti-glaucoma surgery. Mann Whitney U test was used to compare number of anti-glaucoma medications (pre- vs post- operative or modified vs control group)

**Fig. 5:**
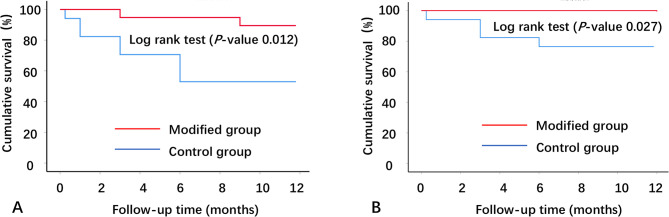
Kaplan–Meier curve for the complete and qualified success of the two groups.**A** The 1-year complete success (criterion 2, with postoperative month 12 mdIOP ≤ 18 mmHg) showing significant difference between the two survival curves of modified and control groups (log rank test, P = 0.012); **B** The 1-year qualified success (criterion 2) also showing significant difference (log rank test, P = 0.027)

Early postoperative IOP was comparable between groups at 1 week (14.22 ± 5.20 vs. 12.97 ± 3.50 mmHg, *P* = 0.318) and 1 month (13.66 ± 7.30 vs. 14.43 ± 2.88 mmHg, *P* = 0.603). From 3 months onward, the study group demonstrated significantly lower IOP than controls: 3 months (11.66 ± 2.84 vs. 16.47 ± 2.75 mmHg), 6 months (11.39 ± 2.82 vs. 16.43 ± 2.79 mmHg), and 12 months (12.38 ± 2.60 vs. 16.82 ± 2.36 mmHg; all *P* < 0.001) (Fig. 3). Medication burden at 12 months also favored the study group (0.11 ± 0.32 vs. 0.43 ± 0.68 medications, *P* = 0.044) (Fig. 4). Survival analysis revealed superior complete (log-rank *P* = 0.012) and qualified (log-rank *P* = 0.027) success rates (mdIOP ≤ 18 mmHg) for the study group (Figs. 5 A-B).

The study group’s safety profile showed several expected postoperative events: hyphema (4 eyes, 21.1%) resolving spontaneously within 10 days; grade 1 shallow AC (4 eyes, 21.1%) improving within 1 week of cycloplegic therapy; and transient IOP elevation (5 eyes, 26.3%) - with 4 cases occurring within the first week (2 managed medically, 2 requiring suture adjustment) and 1 at 2 months (responding to ocular massage). Two cases (10.5%) developed malignant glaucoma refractory to medical management, ultimately requiring surgical intervention (phacoemulsification with vitrectomy) with subsequent IOP control. One eye (5.3%) presented with exudative choroidal detachment that resolved completely with intravenous mannitol, atropine, and peribulbar triamcinolone therapy (Table 3).

**Table 3:** The postoperative complications of modified SAPC vs. compound trabeculectomy in treating PACG within 1 year

Postoperative complications	Grouping, No. (%)		*P* value
	Study (*N* = 19)	Control (*N* = 17)	
Postoperative transient ocular hypertension	5 (26.32)	1 (5.88)	0.232
>21 mmHg and ≤ 30 mmHg	1 (5.26)	1 (5.88)	1.000
>30 mmHg	4 (21.05)	0 (0)	0.140
Hyphema	4 (21.05)	2 (11.76)	0.765
Shallow anterior chamber (grade 1)	4 (21.05)	2 (11.76)	0.765
Malignant glaucoma	2 (10.53)	0 (0)	0.487
Choroidal detachment	1 (5.26)	1 (5.88)	1.000
Transient hypotony	0 (0)	0 (0)	-

*PACG* Primary angle-closure glaucoma, *SAPC S*uture-assisted penetrating canaloplasty

*P* < 0.05 was considered to be statistically significant

Long-term follow-up revealed distinct bleb characteristics between the surgical approaches. No filtering blebs formed in the study group. Between 6 and 12 months post-SAPC, five eyes (26.3%) from four patients subsequently underwent phacoemulsification with intraocular lens implantation. Notably, no hypotony occurred in either group. Comparative analysis of complication rates demonstrated no statistically significant differences between groups (Table 3).

## Discussion

Over the past decade, bleb-independent canaloplasty has gained increasing recognition among glaucoma specialists as an innovative surgical approach that circumvents the primary limitations of compound trabeculectomy and glaucoma drainage devices, particularly subconjunctival scarring and bleb-related complications[4]. This technique works by reconstructing the physiological aqueous outflow pathway through suture-mediated dilation of SC. The pioneering work of Lewis et al. in 2007 first demonstrated the IOP-lowering effectiveness of canaloplasty in patients with POAG, with subsequent studies suggesting that the tensioning suture itself may be the key mechanism underlying IOP reduction in ab externo SC surgery.[10–14]

Non-penetrating canaloplasty is primarily limited to POAG and unsuitable for PACG[15–17], which is highly prevalent in China. To bridge this gap, Liang Y et al. developed penetrating canaloplasty, reporting a 75.9% complete success rate at one year in challenging cases like glaucoma secondary to iridocorneal endothelial syndrome.[1, 2] Another practical barrier to widespread canaloplasty adoption has been the prohibitive cost of disposable iTrack microcatheters. Recent innovations using prefabricated double-helix 6 − 0 polypropylene sutures as catheter substitutes have shown comparable effectiveness to microcatheter-assisted procedures in both surgical feasibility and postoperative outcomes, offering a more accessible alternative[3, 17, 18].

Other minimally invasive glaucoma surgery (MIGS) techniques—such as trabecular stents (e.g., iStent, Hydrus), ablation devices (e.g., Trabectome), goniotomy, suprachoroidal shunts, endoscopic cyclophotocoagulation, and subconjunctival implants (e.g., Xen)—provide safer and more effective options than compound trabeculectomy, typically achieving 15–50% IOP reductions with a favorable safety profile (e.g., hyphema ≤ 20%, hypotony ≤ 15.4%). [19] However, most MIGS devices (e.g., iStent, Hydrus, Trabectome) require an open ACA and are therefore primarily used in POAG. [19] For PACG, phacoemulsification is often necessary first to deepen the AC and widen the ACA, creating suitable anatomical conditions for MIGS device implantation.

Building upon previous research, we implemented two key modifications to the SAPC technique that significantly enhanced its clinical performance. First, we innovatively replaced microcatheters by thermoforming the tip of a 5 − 0 polypropylene suture into a blunt spherical shape. This modification not only maintained sufficient elasticity without requiring a double-helix structure but also achieved dual benefits: reducing operative time by approximately 30% and decreasing consumable costs by over 60%. Most notably, this approach yielded an 87.5% success rate for 360° catheterization, surpassing the 82.9% reported in prior studies[2]. Second, we incorporated two prefabricated adjustable sutures specifically designed to mitigate early postoperative IOP - a well-documented complication of non-penetrating canaloplasty that can compromise optic nerve function and patient satisfaction[1].

Our results demonstrated SAPC’s superior IOP-lowering effectiveness at 12-month follow-up, reducing mdIOP from 21.96 ± 6.00 mmHg preoperatively to 12.38 ± 2.60 mmHg (*P*< 0.001). This performance exceeded both our control group (16.82 ± 2.36 mmHg) and published outcomes for standard penetrating canaloplasty (16.6 ± 5.3 mmHg)[2]. The medication burden similarly decreased from 3.21 ± 1.03 to 0.11 ± 0.32 agents, outperforming both control subjects (0.43 ± 0.68) and literature benchmarks (0.2 ± 0.6)[2]. Remarkably, we achieved 100% complete success at 1-year follow-up versus 75.9% in comparable studies[2], potentially attributable to our optimized suture adjustment protocol based on individual postoperative IOP responses.

Interestingly, while preoperative mdIOP was higher in the study group, both techniques showed comparable early pressure control at 1-week (14.22 ± 5.20 vs. 12.97 ± 3.50 mmHg, *P* = 0.318) and 1-month (13.66 ± 7.30 vs. 14.43 ± 2.88 mmHg, *P* = 0.603) intervals. The SAPC group’s distinct “L-shaped” pressure curve (versus the “V-shaped” trajectory of trabeculectomy) emerged between 1-week and 1-month postoperatively, ultimately achieving superior long-term pressure reduction despite initially higher baseline IOP (Fig. 3). This temporal pattern suggests SAPC’s mechanism involves gradual but sustained outflow enhancement rather than the acute filtration characteristic of trabeculectomy.

Our study revealed important insights into the safety profile of SAPC in PACG patients. The most frequent complication was transient postoperative IOP, occurring in 5 eyes (26.32%) - a rate notably lower than the 37.8% reported in comparable studies[1]. Four of these cases emerged within the first postoperative week and were successfully managed through medical therapy (2 eyes), AC paracentesis (1 eye), or adjustable suture removal (1 eye), achieving IOP below 20 mmHg. This early pressure elevation likely reflects incomplete SC dilation immediately following surgery. A 26.32% rate of transient IOP elevation, while manageable, is substantial. This underscores that SAPC is not a zero-risk procedure and requires careful postoperative monitoring and readiness for intervention (e.g., suture adjustment). This rate may limit its applicability in settings without close follow-up capabilities. Hyphema developed in 4 cases (21.05%), resolving spontaneously within 10 days without compromising long-term pressure control. While our hyphema incidence exceeded the 15.8% reported with microcatheter-assisted techniques[10], this difference may stem from the 5 − 0 polypropylene suture’s slightly greater surface roughness compared to iTrack microcatheters, combined with our inability to administer viscoelastic during suture threading[19–22]. These factors potentially increased microtrauma to the SC during the catheterization process.

The incidence of grade 1 AC shallowing (21.05%) mirrored that observed in the compound trabeculectomy group (10.00%), with no cases progressing to hypotony (IOP < 5 mmHg). This phenomenon likely reflects a balance between two competing mechanisms: the watertight scleral flap closure versus enhanced early filtration due to MMC application[23] and selective suture adjustment. Notably, we encountered two cases of malignant glaucoma (10.53%) - both in younger patients (ages 33 and 40) with short axial lengths (< 22 mm) and ciliary body anterior rotation confirmed by preoperative UBM. This incidence exceeds both our control group and literature reports (2–4%)[24], suggesting SAPC may pose particular risks for this patient subset. Importantly, we observed no severe short-term complications like massive hyphema (> 1/2 AC volume), Descemet’s membrane rupture, or suprachoroidal hemorrhage. However, longer follow-up remains necessary to assess potential late-onset concerns such as suture exposure or progressive endothelial cell loss.

Our postoperative observations demonstrated that SAPC combined with adjustable sutures was effective in reducing mdIOP in patients with PACG, exhibiting a broad range of indications. This technique is particularly advantageous for mild-to-moderate PACG, offering superior IOP reduction, avoidance of specialized consumables, and lower procedural costs, thereby expanding treatment options for both ophthalmologists and patients. Nevertheless, this study has several limitations.

On the one hand, as a retrospective, non-randomized clinical trial, the findings may be subject to selection bias. While the groups were comparable in key preoperative metrics (IOP, medications), the temporal assignment (control group earlier, study group later) means unmeasured factors like evolving surgical techniques or patient selection criteria could influence outcomes. This bias potentially inflates the perceived efficacy of SAPC. The results should therefore be interpreted as promising preliminary evidence of superiority, necessitating validation through a prospective randomized controlled trial. On the other hand, the stringent inclusion criteria resulted in a limited cohort of only 17 PACG patients (24 eyes) over a one-year screening period. The higher rate of malignant glaucoma in the study group (10.5% vs. 0%) did not reach statistical significance due to the small sample size, but it remains a clinically alarming finding. The small sample size is the primary limitation of this study, leading to low statistical power, especially for complication analysis. Similarly, a prospective randomized controlled trial with a larger, calculated sample size is the necessary next step to confirm these findings. Furthermore, this study did not incorporate quantitative perimetric measures such as visual field mean deviation, nor did it utilize detailed BCVA metrics. Although BCVA was reported in broad categories, the absence of functional visual field data constrains a more nuanced understanding of the procedure’s influence on disease progression and visual function in PACG. Future investigations should include standardized perimetry and continuous BCVA measurements to allow for a more comprehensive assessment of surgical outcomes.

To mitigate statistical errors associated with the small sample size, we included both eyes of the same patient in the analysis. However, including both eyes from the same patient (5 patients in the SAPC group) violates the assumption of independence for statistical tests and can introduce correlation bias. Additionally, the study group had a significantly younger mean age compared to the control group (57.53 ± 12.25 vs. 68.50 ± 7.22, *P* < 0.001), suggesting that this procedure may be particularly suitable for younger patients with compromised corneal endothelial function who are at high risk from phacoemulsification or are not candidates for lens removal. Although the age difference was not statistically significant (*P* = 0.063), the clinical difference (mean 6 years younger in the SAPC group) is notable. Younger patients may have more robust wound healing responses, which could theoretically favor the bleb-independent SAPC technique over trabeculectomy, which is prone to scarring. This demographic discrepancy is a confounder that could skew the results in favor of SAPC. Notably, the 10.53% rate of malignant glaucoma is indeed concerning and higher than literature benchmarks. For younger PACG patients with short axial lengths and anteriorly rotated ciliary bodies, SAPC may pose a specific risk by deeply manipulating the angle and ciliary body region. We recommend that these factors be absolute key considerations in patient selection and risk stratification for this procedure. Furthermore, the one-year follow-up period is adequate for evaluating procedural feasibility and short-term efficacy; however, it remains insufficient to assess long-term safety issues such as suture degradation, late-onset fibrosis, or progressive endothelial cell loss. It should be emphasized that the present findings reflect short-term outcomes, and studies with extended follow-up durations are essential. 

## Data Availability

The data supporting the findings of this study are available upon request from the corresponding author [SS].

## Abbreviations

AC: anterior chamber
ACA: anterior chamber angle
AS-OCT: anterior segment optical coherence tomography
BCVA: best-corrected visual acuity
HM: hand motion
IOP: intraocular pressure
mdIOP: the mean diurnal intraocular pressure
MIGS: minimally invasive glaucoma surgery
PACG: primary angle-closure glaucoma
POAG: primary open angle glaucoma
SAPC: suture-assisted penetrating canaloplasty
SC: Schlemm’s canal
UBM: ultrasound biomicroscopy
